# Two Waves of COVID-19 in University Setting: Mental Health and Underlying Risk Factors

**DOI:** 10.3389/fpsyg.2021.780071

**Published:** 2021-12-22

**Authors:** Lucie Křeménková, Jan Sebastian Novotný, Jana Kvintová

**Affiliations:** ^1^Department of Psychology and Abnormal Psychology, Faculty of Education, Palacký University Olomouc, Olomouc, Czechia; ^2^Translational Neuroscience and Aging Program, Centre for Translational Medicine, International Clinical Research Centre, St. Anne’s University Hospital Brno, Brno, Czechia

**Keywords:** COVID-19, pandemic, mental health, university setting, risk factors

## Abstract

The aim of the paper was to assess the differences in the mental distress of university students in the first and second waves of COVID-19, to compare these levels with that of the general population as well as to identify the risk factors associated with the changes in mental health. A total of 2,025 university students in core psychology courses in all years of study at the Faculty of Education at Palacký University Olomouc were approached *via* e-mail. Of this number of students, 800 students took part in the study, divided into two groups from the spring (*N* = 438) and autumn (*N* = 362) pandemic waves. The data were collected online *via* Google Forms using a battery of questionnaires and analyzed using the Wilcoxon–Mann–Whitney test, One-Sample Wilcoxon Signed Rank Test and binary logistic regression. The results showed a high prevalence of depressive symptoms (38.4 and 51.4%), significant anxiety (43.8 and 37%), and high stress (19.9 and 22.9%) among students in both waves of the pandemic. Depression and stress also increased significantly during the second wave compared with the first one (*r* = 0.18 [0.12, 0.25] and *r* = 0.08 [0.01, 0.14]). Finally, university students showed significantly higher levels of mental distress than the general population in all of the variables and in both waves (*r* = 0.42–0.86). A variety of factors influenced different aspects of mental distress in the spring and autumn pandemic waves. Emotion regulation emerged as the most significant and pervasive factor, both influencing all of the three indicators of mental distress and being a significant predictor in both waves.

## Introduction

The COVID-19 pandemic, which began in early 2020, has caused huge changes in the way societies function and affected the way almost everyone lives. In the year and a half of its duration, it has spread all over the world and different countries have experienced one or more waves of increase in the number of COVID-19 cases, which has forced them to apply various restrictions and epidemic measures to combat the spread of the pandemic. These measures have affected many areas of life such as travel, social interaction, shopping, education, health care delivery and more. In addition, the repeated local suppression of the epidemic, coupled with the relaxation of measures and the simultaneous emergence of new mutations of the coronavirus (Torjesen, 2021) which again increase the spread of the virus and lead to the reintroduction of measures creates a state of uncertainty with little possibility of predicting future developments. This multitude of changes, the fear of COVID-19 and the uncertainty about the future course of the pandemic also has a significant negative impact on the mental health of the population, as confirmed by a number of studies (e.g., Lakhan et al., 2020; Novotný et al., 2020; Rossi et al., 2020; Bueno-Notivol et al., 2021; Wang et al., 2021). The impact on mental health is also related to the degree of personal experience of the pandemic and how much change the pandemic and the restrictions have caused in personal life.

One group that has been greatly affected by the COVID-19 pandemic and its consequences is university students. They have had to cope not only with the general societal changes but also with the disruption of their studies. The closure of schools, the banning of face-to-face tuition and the rapid transition to distance learning, including the resulting uncertainty about the future course of their own studies (and, figuratively, concerns about future professional life, especially for graduating classes) as well as the mandated work obligation not only pose practical challenges for everyday life but also create increased pressure on mental health. Increased mental distress is then risky not only because of the disruption to mental health itself but also because of its potential long-term negative impact on further studies and on everyday functioning in general.

A number of studies have already addressed the impact of the COVID-19 pandemic on students’ lives and mental health, confirming the negative impact of the pandemic (e.g., Son et al., 2020; Browning et al., 2021; Fruehwirth et al., 2021; Křeménková and Novotný, 2021; Villani et al., 2021; Xu et al., 2021). But although a number of findings and observations are available, much is still unknown. At the same time, not all of the findings are easily transferable globally, as students in each country live in different conditions, are affected by different pandemic progression, disruptions in society as well as limitations to their studies. Meanwhile, understanding properly how the COVID-19 pandemic affects students’ mental health in a given context and which factors represent risk or protective influences is crucial for providing effective help to students in their studies and on a personal basis. The aim of this paper was therefore to examine the changes in the mental health of university students during the first and second waves of the COVID-19 pandemic in the Czechia, to compare the level of mental distress in students with the general population and to identify the factors that influence the presence of depression, anxiety and stress in each wave of the pandemic.

## Materials and Methods

### Study Design and Sample

The study was conducted in two phases. The first data collection took place from April 8 to April 30, 2020. This period corresponded to the first wave of the COVID-19 pandemic in the Czechia and the summer^1^ semester of the 2019/2020 academic year. The second data collection took place from October 30 to November 30, 2020. This period coincided with the middle of the winter semester of the 2020/2021 academic year and the peak of the second wave of the COVID-19 pandemic in the Czechia. In addition to a number of strict epidemic measures imposed in the Czechia limiting normal daily functioning and social interaction, both data collection periods saw the long-term closure of all schools, including universities (in the second wave, universities were closed for almost the entire academic year 2020/2021) and students participated in compulsory online classes.

A total of 1,053 (wave 1) and 972 (wave 2) students of the Faculty of Education at Palacký University Olomouc in Olomouc were invited to participate in the study *via* their university emails (students were required to check their email regularly). This included all students in core psychology courses in all years of study and covered core students of the Faculty of Education as well as a minority of students from other faculties of Palacký University Olomouc attending courses at the Faculty of Education as part of their study. 438 (41.6%) students in wave 1 and 362 (37.2%) students in wave 2 participated in the study. To compare mental distress between university students and the general population, partial data (youngest group aged 24–40, *N* = 265) from the population-based epidemiological cohort from a Kardiovize study (Novotný et al., 2020; unpublished data for wave 2) were used.

### Measure and Instruments

The data were collected using a Google Forms online survey consisting of several parts. The first part contained demographic questions, items regarding the COVID-19 pandemic and government measures concerning life and study (measured on a 5-point Likert-type scale ranging the magnitude of the negative effect), related concerns about family members’ health and about finishing the semester (measured on a 5-point Likert-type scale ranging from “totally disagree” to “total agree”) as well as the perceptions concerning the university’s approach and communication (measured on a 5-point Likert-type scale ranging from “totally disagree” to “totally agree” and by means of dichotomic yes/no items).

The second part consisted of a battery of 6 psychological tools. The Patient Health Questionnaire (PHQ-4; Löwe et al., 2010) is short four-item tool with a 4-point Likert-type scale measuring the severity of depressive symptoms and anxiety (scoring 0–6 for depressive symptoms and anxiety). A score of three or more represented the presence of depressive and anxiety symptoms. The Perceived Stress Scale (PSS-4; Cohen and Williamson, 1988) is a short four-item uni-dimensional tool with a 5-point Likert-type scale measuring stress levels (scoring 0–16). A score of 11 or more represented the presence of high stress. The Prosocial Behavioral Intentions Scale (PBIS; Baumsteiger and Siegel, 2019) is a four-item uni-dimensional tool with a 7-point Likert-type scale measuring the levels of intention to behave prosocially (scoring 4–28). The Internal External Locus of Control-4 scale (IE-4; Kovaleva, 2012) is a four-item uni-dimensional tool with a 5-point Likert-type scale measuring the presence of internal and external locus of control (scoring 1–5). The Connor-Davidson Brief Resilience Scale (CD-RISC; Vaishnavi et al., 2007) is a two-item uni-dimensional tool with a 5-point Likert-type scale measuring resilience (scoring 0–8). The Difficulties in Emotion Regulation Scale (DERS; Gratz and Roemer, 2004) is a 18-item tool with a 5-point Likert-type scale measuring the presence of the seven types of emotion-related difficulties (subscale scoring 3–15, total scale scoring 18–90). The following three indicators of COVID-19 impact on psychological well-being were used: severity of depressive symptoms and anxiety level (as subscales of PHQ-4) and stress level (as measured by PSS-4). The presence of significant depressive symptoms and anxiety was defined as a PHQ score equal to or greater than 3, while the presence of high levels of stress was defined as a PSS score greater than 10.

### Data Analysis

Descriptive statistics were performed on demographic variables. Mann-Whitney test with r effect size calculation was performed to compare mental distress across waves. One-Sample Wilcoxon Signed Rank Test with continuity correction was used to compare mental distress in university students and the general population. A series of binary logistic analyses were used to assess the effect of other factors on mental distress. The binary variables of presence of depressive and anxiety symptoms and high stress were used as outcomes. Any two-sided *P* < 0.05 was considered statistically significant. Statistical analyses and data visualizations were performed R v.4.1.1^2^ using BSDA (v.1.2.1), coin (v.1.4-2), dplyr (v.1.0.7), ggplot2 (v.3.3.5), ggpubr (v.0.4.0), rcompanion (v.2.4.1), rstatix (v.0.7.0), and stats (v.4.1.1) packages.

### Ethical Consideration

All participants were informed of the confidentiality of their answers and signed an online informed consent form prior to the completion of the questionnaire. No specific information enabling the identification of specific students (such as IP address, student name or ID number, specific field of study, etc.) was obtained as part of the online data collection. The research protocol of the study was approved by the Ethics Committee of the Faculty of Education.

## Results

### Sample Demographics

The study population consisted of 800 university students, 438 in wave 1 (mean age = 23.8 ± 7, 408 [93.2%] women) and 362 in wave 2 (mean age = 26.6 ± 9.8, 342 [94.5%] women). This sex ratio acceptably corresponded to the distribution of students at the Faculty of Education. The majority of participants were in the non-graduate year (393 [89.7] and 302 [83.4%]) and were full-time students (354 [80.8] and 318 [60.2%]).

### Mental Distress During Two Waves of the COVID-19 Pandemic

The prevalence of significant symptoms of each indicator of mental distress in the whole sample in the spring and autumn waves was 38.4 and 51.4% for depression, 43.8 and 37% for anxiety and slightly lower 19.9 and 22.9% for stress. This prevalence was similar for both sexes in the first wave but in the second wave the prevalence of mental distress was noticeably lower in men than in women (2.5–4 times). In the context of the type of study, the prevalence of mental distress was approximately half as high in distance learners compared with full-time students concerning most variables, especially in the second wave (Table 1).

**TABLE 1:** Mean scores ± SD and prevalence of mental distress in waves 1 and 2.

	Depression	Anxiety	Stress
**Wave 1 (Spring)**			
Mean score ± SD	2.18 ± 1.74	2.29 ± 1.6	7.37 ± 3.39
*Males*	1.9 ± 2.47	1.4 ± 1.59	6.0 ± 5.04
*Females*	2.2 ± 1.68	2.36 ± 1.58	7.47 ± 3.22
*Full-time study*	2.16 ± 1.72	2.39 ± 1.62	7.46 ± 3.34
*Distant learning*	2.25 ± 1.86	1.89 ± 1.46	7.0 ± 3.59
Prevalence of significant mental distress (N [%])	168 [38.4%]	192 [43.8%]	87 [19.9%]
*Males*	12 [40%]	12 [40%]	6 [20%]
*Females*	156 [38.2%]	180 [44.1%]	81 [19.9%]
*Full-time study*	132 [37.3%]	168 [47.5%]	78 [22%]
*Distant learning*	36 [42.9%]	24 [28.6%]	9 [10.7%]
**Wave 2 (Autumn)**			
Mean score ± SD	2.77 ± 1.63	2.13 ± 1.67	7.9 ± 3.29
*Males*	1.7 ± 1.45	1.2 ± 0.89	4.5 ± 2.44
*Females*	2.83 ± 1.62	2.19 ± 1.68	8.09 ± 3.23
*Full-time study*	3.17 ± 1.59	2.34 ± 1.77	8.9 ± 3.12
*Distant learning*	2.17 ± 1.51	1.82 ± 1.45	6.38 ± 2.96
Prevalence of significant mental distress (N [%])	186 [51.4%]	134 [37%]	83 [22.9%]
*Males*	4 [20%]	2 [10%]	1 [5%]
*Females*	182 [53.2%]	132 [38.6%]	82 [22.6%]
*Full-time study*	134 [61.5%]	96 [44%]	78 [35.8%]
*Distant learning*	52 [36.1%]	38 [26.4%]	5 [3.5%]

Comparisons across the pandemic waves revealed that depressive symptoms (*P* < 0.001, *r* = 0.18 [95% CI = 0.12, 0.25]) and stress levels (*P* = 0.032, *r* = 0.08 [95% CI = 0.01, 0.14]) increased in the second wave compared with the first wave, while anxiety levels remained the same (*P* = 0.072). To control for the possible effect of a general atypical ratio of the two sexes and a slightly different ratio of full-time and distance students between the two waves, a detailed analysis was performed for each subset. In the context of sex, differences between the two waves of the pandemic were only found in women, both in depressive symptoms (*P* < 0.001, *r* = 0.20 [95% CI = 0.12, 0.27]) and stress levels (*P* = 0.011, *r* = 0.09 [95% CI = 0.03, 0.17]) but not in anxiety (*P* = 0.062). Men did not show any differences in mental distress between the waves (*P* = 0.71, 0.81, and 0.64 for depressive symptoms, anxiety and stress, respectively). In terms of the type of study, the results showed differences between the two waves only in full-time students for depressive symptoms (*P* < 0.001, *r* = 0.29 [95% CI = 0.22, 0.36]) and stress levels (*P* < 0.001, *r* = 0.20 [95% CI = 0.12, 0.27]), while anxiety did not differ between the two waves (*P* = 0.51). Similarly, there were no differences in depressive symptoms (*P* = 0.98), anxiety (*P* = 0.52) or stress levels (*P* = 0.36) in distance learning students.

### Differences in Mental Distress Between University Students and the General Population

**FIGURE 1:**
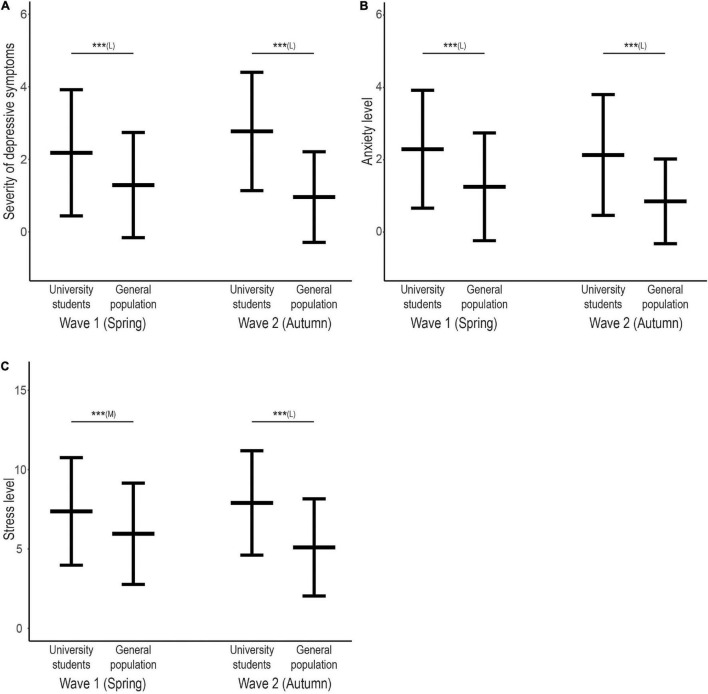
Levels of mental distress in university students and the general population in each wave of the COVID-19 pandemic. Line graphs showing mean score and ± 1 Standard Deviation range for **(A)** severity of depressive symptoms, **(B)** anxiety levels, and **(C)** stress levels. Upper horizontal bars indicate significant differences in mental distress outcomes between university students and the general population in a particular pandemic wave (***P < 0.01), letters indicate effect size (M = moderate, L = large).

A comparison of the individual indicators of mental distress between university students and the general population showed that in both pandemic waves university students experienced significantly stronger depressive symptoms (*r* = 0.54 and 0.86, respectively), levels of anxiety (*r* = 0.64 and 0.86, respectively) and perceived stress (*r* = 0.42 and 0.79, respectively) (Figure 1 and Table 2). This substantially higher mental distress was also observed in all subgroups by sex and type of study, except for depressive symptoms (first spring wave) and stress levels (both pandemic waves) in men (Supplementary Table 1).

**TABLE 2:** Comparison of mental distress in university students and the general population.

	Present study mean ± SD	Kardiovize study (24–40 years) mean ± SD	*P*	95% CI	r^a^
**Wave 1 (spring)**					
Depression	2.18 ± 1.74	1.29 ± 1.45	<0.001	[2.500, 3.000]	0.54 (L)
Anxiety	2.29 ± 1.6	1.25 ± 1.49	<0.001	[2.499, 2.500]	0.64 (L)
Stress	7.37 ± 3.39	5.96 ± 3.19	<0.001	[7.000, 7.500]	0.42 (M)
**Wave 2 (autumn)**					
Depression	2.77 ± 1.63	0.96 ± 1.25	<0.001	[2.999, 3.000]	0.86 (L)
Anxiety	2.13 ± 1.67	0.85 ± 1.17	<0.001	[2.499, 2.500]	0.86 (L)
Stress	7.9 ± 3.29	5.10 ± 3.06	<0.001	[7.500, 8.499]	0.79 (L)

*^a^Letters indicate the size of the effect (M–moderate, L–large).*

### Risk Factors Associated With Increased Depressive Symptoms

**FIGURE 2:**
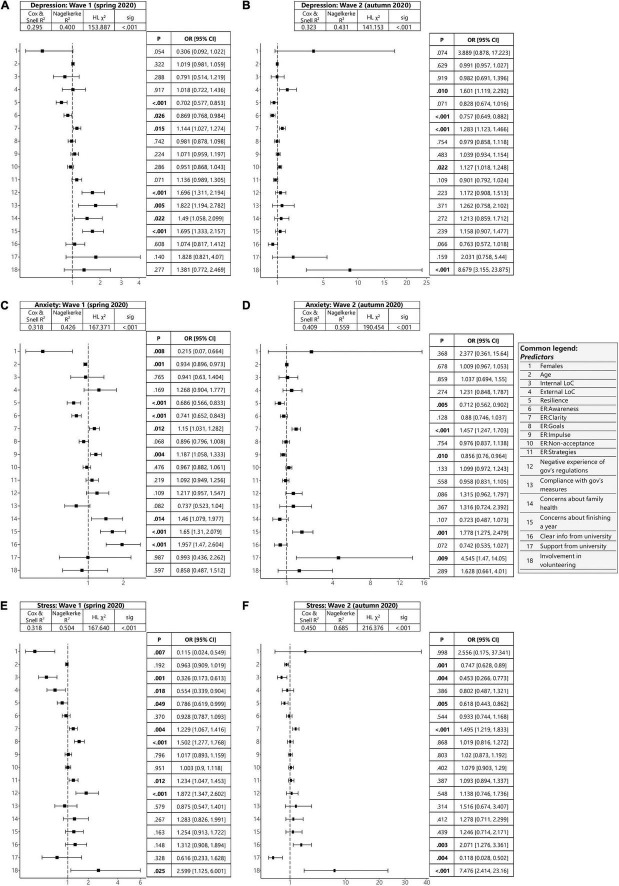
Binomial logistic regression model assessing the association between increased mental distress and risk factors. Forest plots of odds ratios with indicated model characteristics and *P*-values plus odds ratios with confidence intervals for each predictor showing **(A)** predictors of depressive symptoms in wave 1, **(B)** predictors of depressive symptoms in wave 2, **(C)** predictors of anxiety symptoms in wave 1, **(D)** predictors of anxiety symptoms in wave 2, **(E)** predictors of high stress in wave 1, and **(F)** predictors of high stress in wave 2. The *x*–axes (odds ratio) are plotted using square-root transformation.

A series of logistic regressions revealed a number of effects of individual predictors on mental distress in waves 1 and 2 (Supplementary Table 2). Within depression, two common factors emerged for both waves: a lack of emotional clarity increased the risk of depressive symptoms (OR [95% CI]: 1.14 [1.03, 1.27] and 1.28 [1.12, 1.47]), whereas a lack of emotional awareness decreased the risk of depressive symptoms (OR [95% CI]: 0.87 [0.77, 0.98] and 0.76 [0.65, 0.88]). In terms of effect size, spring compliance with government regulations (OR [95% CI] = 1.82 [1.19, 2.78]), negative perceptions of government actions (OR [95% CI] = 1.70 [1.31, 2.19]) and concerns about completing the year (OR [95% CI] = 1.70 [1.33, 2.16]) appeared to increase the risk of depressive symptoms (Figure 2A). Within the autumn wave, involvement in volunteer activities emerged as the strongest predictor (OR [95% CI] = 8.68 [3.16, 23.88]). Women (OR [95% CI] = 3.89 [0.88, 17.22]) and students who felt supported by the university (OR [95% CI] = 2.03 [0.76, 5.44]) also had higher rates of depressive symptoms (Figure 2B).

### Risk Factors Associated With Increased Anxiety

In the context of anxiety, several factors appeared to increase the risk of anxiety symptoms across both waves. Concerns about completing the year (OR [95% CI]: 1.65 [1.31, 2.08] and 1.78 [1.28, 2.48]) and a lack of emotional clarity (OR [95% CI]: 1.15 [1.03, 1.28] and 1.46 [1.25, 1.70]) were associated with increased the risk of anxiety symptoms across both waves. Impulse control difficulties were associated with increased the risk of anxiety in the spring wave (OR [95% CI] = 1.18 [1.06, 1.33]) but decreased it in the autumn wave (OR [95% CI] = 0.86 [0.76, 0.96]). Finally, higher resilience (OR [95% CI]: 0.68 [0.57, 0.83] and 0.71 [0.56, 0.90], respectively) seemed to be acting as a protective factor against increased anxiety. Students who perceived information from the university as more clear in the spring (OR [95% CI] = 1.96 [1.47, 2.60]) and who were concerned about completing the year (OR [95% CI] = 1.65 [1.31, 2.08]) showed the highest levels of anxiety (Figure 2C). In the spring, women were significantly less anxious than men (OR [95% CI] = 0.22 [0.07, 0.66]), whereas in the autumn they were already more anxious than men (OR [95% CI] = 2.38 [0.36, 15.64]). In conjunction with this, students who felt supported by the university (OR [95% CI] = 4.55 [1.47, 14.05]), were anxious about completing the year (OR [95% CI] = 1.78 [1.28, 2.48]) and were involved in volunteer activities (OR [95% CI] = 1.63 [0.66, 4.01]) were more anxious in the autumn (Figure 2D).

### Risk Factors Associated With Increased Stress

Regarding stress, across both waves, engagement in volunteer activities (OR [95% CI] = 2.60 [1.12, 6.0] and 7.48 [2.41, 23.16]) and a lack of emotional clarity (OR [95% CI] = 1.23 [1.07, 1.42] and 1.50 [1.22, 1. 83]) represent risk factors, whereas greater resilience (OR [95% CI]: 0.79 [0.62, 1.0] and 0.62 [0.44, 0.86]) and greater internal locus of control (OR [95% CI]: 0.33 [0.17, 0.61] and 0.12 [0.03, 0.50]) represent protective factors (Figures 2E,F). In addition to engaging in volunteer activities, the most significant predictors of high stress in the spring were negative perceptions of government action (OR [95% CI] = 1.87 [1.35, 2.60]) and difficulty engaging in goal-oriented behavior (OR [95% CI] = 1.50 [1.28, 1.77]). In contrast, women were at a significantly lower risk of stress in the spring than men (OR [95% CI] = 0.12 [0.02, 0.55]) (Figure 2E). In addition to volunteering, positive perceived awareness from the university appeared to be a risk factor in the autumn wave (OR [95% CI] = 2.07 [1.28, 3.36]). Conversely, perceived support from the university was a significant protective factor against high stress (OR [95% CI] = 0.12 [0.03, 0.50]) (Figure 2F).

## Discussion

The objectives of this study were threefold: first, to analyse the level of mental distress in university students during the first and second waves of COVID-19 in the Czechia; second, to compare the mental distress of students with that of the general population; and third, to identify the factors that might associate with the presence of depression, anxiety and stress in either wave of the pandemic. The findings showed that the COVID-19 pandemic posed a significant risk to the mental health of university students as the prevalence of each type of mental distress in both waves of the pandemic was around one-fifth of the sample for high levels of stress and between one-third and one-half of the sample for depression and anxiety, reaching as many as 51% (over half of the sample) of students showing significant depressive symptoms during the second wave of the pandemic. This prevalence of mental distress is similar to that of healthcare professionals who have been hit hardest by the effects of the COVID-19 pandemic (Kang et al., 2020; Şahin et al., 2020; Guo et al., 2021; Hao et al., 2021). In agreement with previous reports (Di Tella et al., 2020; Holingue et al., 2020; Luo et al., 2020) we also found a more pronounced impact of COVID-19 associated measures on the mental health of women. The lower prevalence of mental distress among students in the distance form of study can be explained by the different characteristics of these two forms of study. For distance learners who are usually already employed while studying the changes and threats to their studies associated with the COVID-19 pandemic do not pose a substantial disruption to their study routine nor do they pose a direct threat to their current employment or future professional career as they do for full-time students (Cao et al., 2020).

Further, we observed that depression and stress increased among students during the second wave of COVID-19 compared with the first spring wave, with anxiety remaining the same, which is similar to previous findings in another directly affected group—healthcare professionals (Magnavita et al., 2021b). This increase is likely influenced by repeated experiences of disruption in the course of study. During the first wave, students were coping with an unfamiliar situation and an unexpected transition to the online environment, which increased their level of mental distress. However, this was a new situation and the tranquil course of the pandemic (with low infection rates) created the impression that this study disruption was only a one-off. The arrival of the second wave in the autumn and the re-closure of universities was thus perceived more negatively by students in the context of previous experiences. Students thus already knew what problems they could expect and it was the second disruption to their studies in a short space of time. The course of the pandemic in the Czechia was also more dramatic with a rapid increase in the number of new cases, supporting the assumption that the second university closure would be longer and the disruption to studies would be more intense. These findings are consistent with previous studies (Nurunnabi et al., 2021; Rudenstine et al., 2021; Viner et al., 2021; Zheng et al., 2021), however, the knowledge of the impact of repeated disruption is still limited.

A both intriguing and concerning finding was the significantly higher level of mental distress of university students compared with the general population that was present across both waves of COVID-19. The reasons for this difference may be several. The younger population in general appears to be more negatively affected by the COVID-19 pandemic (Novotný et al., 2020; Gasteiger et al., 2021; Simon et al., 2021; Varma et al., 2021). This may be due to the association of greater worries about studies, job security and financial stability with younger age, and richer life experiences and reduced life expectations in the older (Roberts et al., 2018; Cao et al., 2020; Russo and Terraneo, 2020). University students (and students in general) also have a much more pronounced direct experience of the effects of the COVID-19 pandemic (similar to health professionals) as the pandemic has affected the key element of their lives compared with the general population. Consistent with this is the fact that unlike the general population where the rates of mental distress decreased slightly in the second wave of the pandemic, they remained the same or even increased in university students, underscoring the devastating impact of the direct and widespread negative experiences of the pandemic on mental health (Magnavita et al., 2021a). However, it is worth noting that although the general population sample was selected to be as similar as possible to the sample of university students, the two samples were understandably somewhat different in their characteristics and composition, including for example the different sex proportions. On the other hand, a detailed analysis of the individual subsets (by sex and type of study) indicated that the observed differences between university students and the general population were indeed universal. Either way, these findings highlight the need to pay attention to the mental health of university students as a group at a greater risk.

An analysis of the factors associated with the presence of mental distress symptoms yielded a number of individual findings that confirmed our previously reported findings (Křeménková and Novotný, 2021). In general, the pre-dominant association with the emotional regulation emerged. In most cases, emotional regulation problems were associated with a greater risk of experiencing mental distress but in some cases they were a protective factor or their effect changed over time. The impairment of mental health associated with emotion regulation problems is a previously described phenomenon in which the lack of ability to control and direct one’s own emotional experience also makes it difficult to control negative emotions triggered by external stimuli (Markarian et al., 2013; Everaert and Joormann, 2019). However, given that repeated negative thinking is associated with depression and anxiety, reduced awareness of one’s own emotions, including negative ones, may protect individuals from their impact on mental health and thus act as a protective factor (Everaert and Joormann, 2019). In the context of regulating the negative emotional impact of a pandemic, the “buffering effect” of resilience as the ability to adapt to adversity, trauma or other major stressors has also been confirmed (Barzilay et al., 2020; Ran et al., 2020). Similarly (in relation to the emotional experiencing of the pandemic), more intense direct experience of other aspects of the COVID-19 pandemic, or the “overloading” of one’s own time resources in the form of volunteering also appears to contribute to a higher presence of mental distress (Mo et al., 2021; Zhang et al., 2021). Finally, support and clear communication and organization by universities, or lack of it, has been shown to contribute to better/worse mental health of students, as already shown in previous studies (Magnavita et al., 2021c).

## Limitations

The study has a few limitations. First, the size of the sample is acceptable, however, the proportion of both sexes is rather imbalanced, although on the whole it corresponds to the proportion of students at the Faculty of Education. The results for men in particular should therefore be viewed with some caution. Second, the research sample is limited to university students of a single faculty with a minority of students from other faculties. A more diverse sample including students from different faculties and disciplines could provide a more generalized data on this issue. Third, the participation rate was below 50% (corresponding, however, to the response rate in the matched epidemiological cohort of Kardiovize). Thus, the willingness to participate in the study and consequently the results may be influenced by the different characteristics and current status of students who chose to participate in the study compared with those who declined to participate (including for example greater willingness to share based on the need to vent their worries, spending more time on the computer, etc.). Finally, the cross-sectional nature of the study did not allow to directly assess the changes in the levels of psychological distress or changes in time. A longitudinal design with multiple time-points for the same respondents might also provide a better insight into the mechanisms of COVID-19 effects on university students.

## Conclusion

In conclusion, the results of this study confirmed our previous findings of high levels of depression, anxiety and stress among university students during the COVID-19 pandemic. A comparison with the general population further highlighted the importance of this topic and the need to pay increased attention not only to the practical aspects of the implementation of the study during the ongoing pandemic, but also to students’ mental health. The provision of psychological help and support needs to be actively promoted both at the individual level (by supporting students in seeking help when needed) and at the institutional level (through university counseling centers).

In this context, the means and procedures for psychological counseling need to be designed or improved. The key tasks in this process should include the increased use of online counseling tools (Renton et al., 2014), the creation/provision of methods to quickly verify the presence of mental distress or increased risk factors as well as support for the development of university counseling centers from the management in terms of creating the time, material and financial conditions for their functioning (Rudnik et al., 2021). This development should be underpinned by the fact that long-term unaddressed depression and stress have a negative impact on mental and psychological health (Houtjes et al., 2014; Belleau et al., 2019), while at the same time most students do not wish to formally address their mental health problems for fear of stigmatization (Ahmedani, 2011; Parcesepe and Cabassa, 2013).

## Data Availability Statement

The original contributions presented in the study are included in the article/Supplementary Material, further inquiries can be directed to the corresponding author.

## Ethics Statement

Ethical review and approval was not required for the study on human participants in accordance with the local legislation and institutional requirements. The patients/participants provided their written informed consent to participate in this study.

## Author Contributions

LK and JN conceived the idea of this study and wrote the draft of the manuscript. JN made the statistical analysis. All authors contributed to the critical revision of the manuscript for important intellectual content and reviewed and approved the submitted version.

## Conflict of Interest

The authors declare that the research was conducted in the absence of any commercial or financial relationships that could be construed as a potential conflict of interest.

## Publisher’s Note

All claims expressed in this article are solely those of the authors and do not necessarily represent those of their affiliated organizations, or those of the publisher, the editors and the reviewers. Any product that may be evaluated in this article, or claim that may be made by its manufacturer, is not guaranteed or endorsed by the publisher.

## Footnotes

1 The academic year in the Czechia is divided into two semesters: winter (September to January) and summer (February to June).

2
https://www.r-project.org/
