# Supplementary material for: Two Waves of COVID-19 in University Setting: Mental Health and Underlying Risk Factors
Source: Front Psychol. 2021 Dec 22;12:780071. doi: 10.3389/fpsyg.2021.780071 (PMC8729183; doi:10.3389/fpsyg.2021.780071)
Supplement: Supplementary file 1 [file Data_Sheet_1.PDF]

Supplementary Material

1 Supplementary Figures and Tables

Supplementary Table S1. Comparison of mental distress in university students and the general population in individual subgroups

	Depression	Anxiety	Stress
Wave 1 (Spring)			
males	0.24	0.64*** (L)	0.22
females	0.57*** (L)	0.67*** (L)	0.34*** (M)
full-time study	0.55*** (L)	0.66*** (L)	0.45*** (M)
distant study	0.51*** (L)	0.52*** (L)	0.32** (M)
Wave 2 (Autumn)			
males	0.81*** (L)	0.85*** (L)	0.06
females	0.86*** (L)	0.85*** (L)	0.67*** (L)
full-time study	0.87*** (L)	0.85*** (L)	0.82*** (L)
distant study	0.85*** (L)	0.86*** (L)	0.48*** (M)

*Effect size values (r) of Wilcoxon signed rank test are shown with asterisks indicating the significance level (**P<.01, ***P<.001) and letters indicating the size of the effect (M – moderate, L – large).*

Supplementary Table S2. Mean scores ±SD of exposures in waves 1 and 2

	Wave 1 (Spring)	Wave 2 (Autumn)
Exposures		
Prosocial behaviour	6.36 ±0.74	6.3 ±0.66
Internal Locus of Control	4.16 ±0.7	3.81 ±0.84
External Locus of Control	2.9 ±0.86	2.72 ±0.85
Resilience	6.09 ±1.47	5.96 ±1.59
Difficulties in emotion regulation		
Total score	46.16 ±10.9	47.13 ±11.58
Lack of emotional awareness	6.21 ±2.21	6.34 ±2.24
Lack of emotional clarity	7.05 ±2.59	7.02 ±2.8
Diffic. engaging in goal-oriented behav.	10.69 ±3.05	11.36 ±2.88
Impulse control difficulties	7.03 ±2.97	7.42 ±3.26
Non-acceptance of emotional response	7.32 ±3.23	6.99 ±3.05
Limited access to emot. regul. strategies	7.87 ±2.97	8 ±3.01
Compliance with epidemic measures	4.52 ±0.71	4.51 ±0.65
Negative perception of gov's measures	2.68 ±1.11	3.09 ±1.16
Concerns about family health	4.32 ±0.89	4.15 ±0.99
Concerns about completing a year	3.79 ±1.24	3.78 ±1.27
Clear info from university	2.92 ±1.03	3.4 ±1.13
Support by university (yes)[a]	381 (87)	314 (86.7)
Involvement in volunteering (yes)[a]	105 (24)	48 (13.3)

[a]*Values presented as no (%).*
